# Supplementary material for: uvCLAP is a fast and non-radioactive method to identify in vivo targets of RNA-binding proteins
Source: Nat Commun. 2018 Mar 20;9:1142. doi: 10.1038/s41467-018-03575-4 (PMC5861125; doi:10.1038/s41467-018-03575-4)
Supplement: Supplementary file 1 — Supplementary Information(PDF 6505 kb) [file 41467_2018_3575_MOESM1_ESM.pdf]

uvCLAP is a fast and non-radioactive method to identify in vivo targets of RNA-binding proteins

Maticzka et al.

Supplementary Figure1

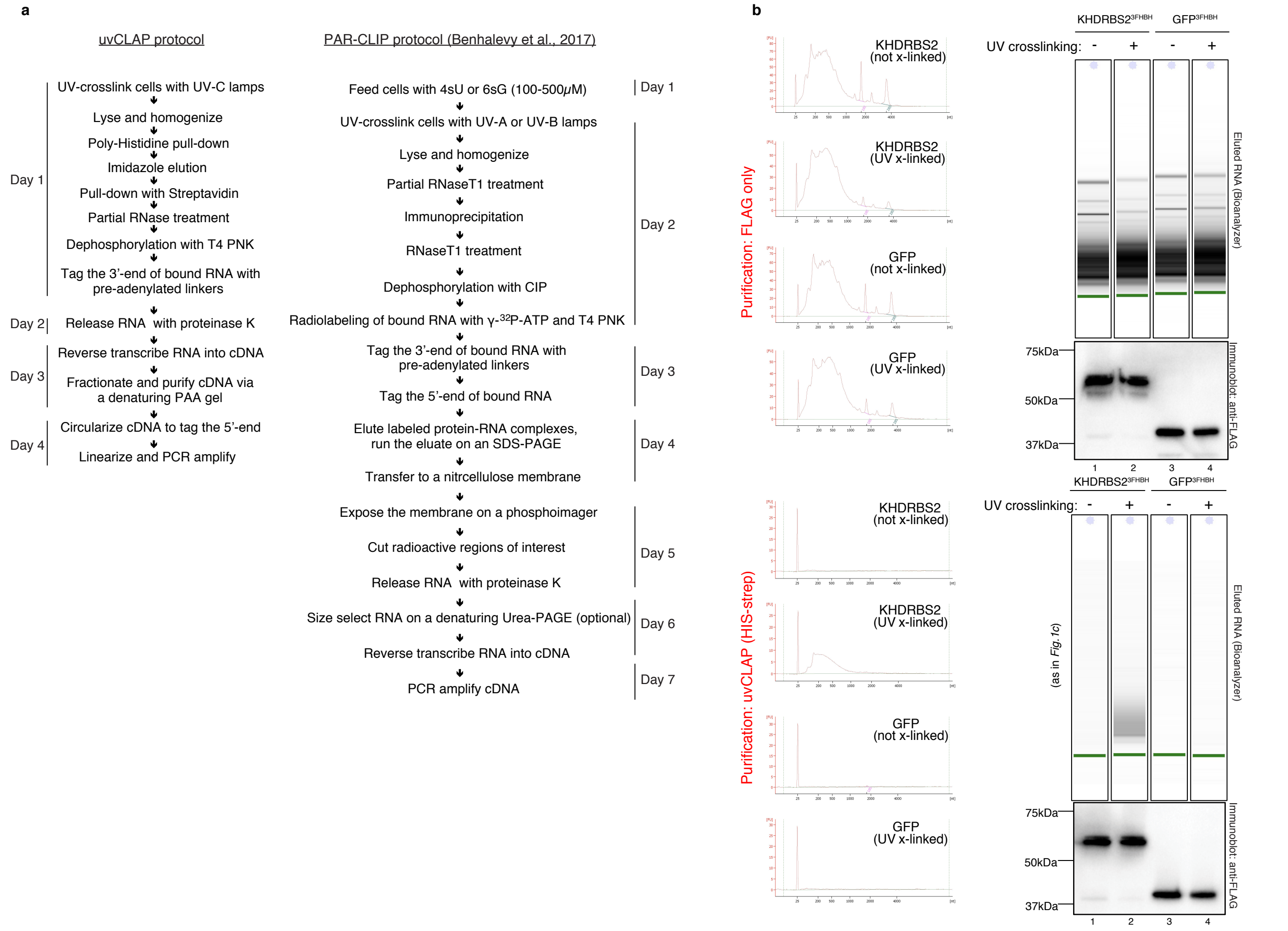

Supplementary Figure 1: **(a)** A step-by-step comparison of the uvCLAP and PAR-CLIP protocols. **(b)** Bioanalyzer traces of RNA purified from non-crosslinked KHDRBS2^{3FHBH} (lane 1), UV-crosslinked KHDRBS2^{3FHBH} (lane 2), non-crosslinked GFP3^{FHBH} (lane 3) and UV-crosslinked GFP3^{FHBH} (lane 4) lysates either via FLAG-beads (top panels) or via the stringent uvCLAP tandem purification scheme as in Fig. 1a (bottom panels). Eluted protein from the same samples, analysed in parallel via immunoblotting with anti-FLAG antibodies are shown below the Bioanalyzer traces.

Supplementary Figure 2

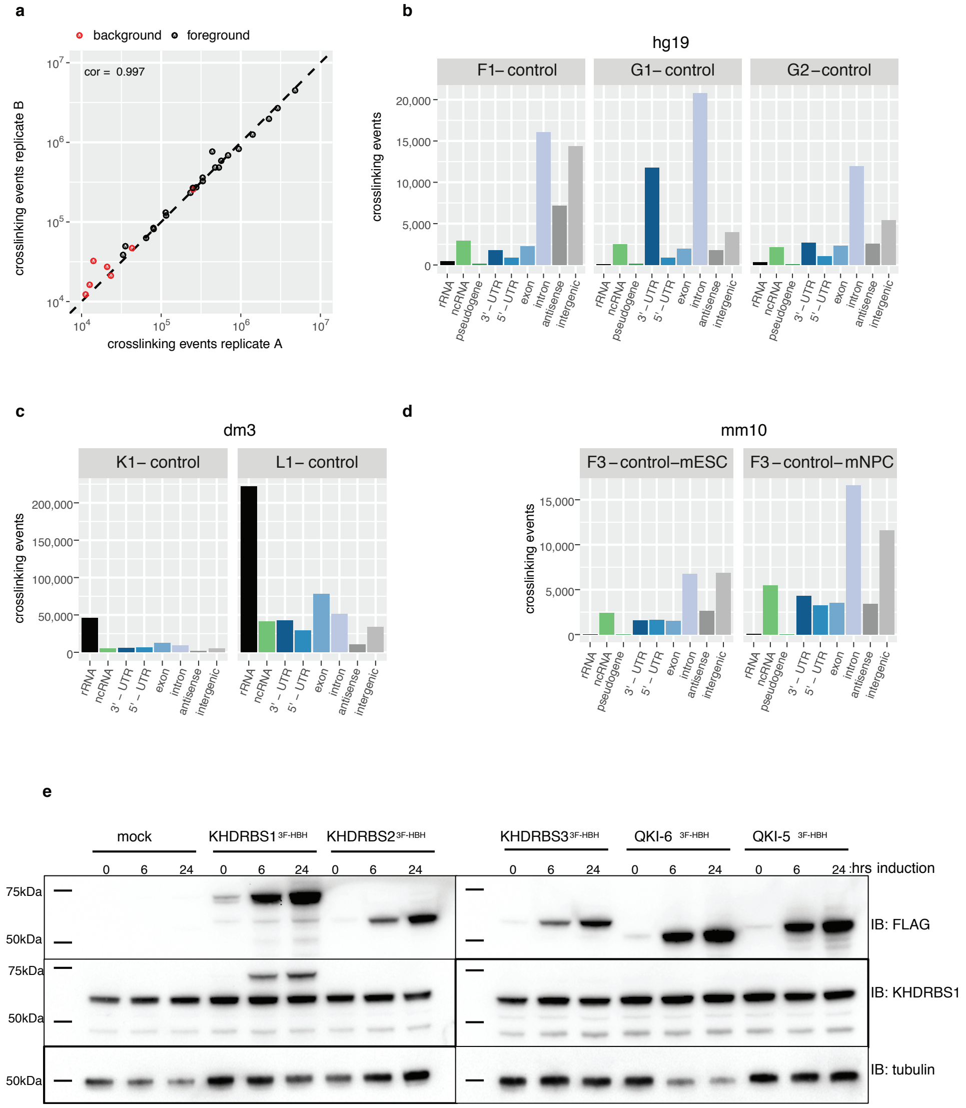

Supplementary Figure 2: **(a)** Expression of stably integrated, 3xFLAG-HBH-tagged KHDRBS1, KHDRBS2, KHDRBS3, QKI-5 and QKI-6 constructs in HEK293 Flp-In T-REX cells. Transgenes were induced with 0.1µg/mL doxycycline for either 6 or 24 hours before lysis and immunoblotting. Immunoblotting with a polyclonal antibody against KHDRBS1 shows that KHDRBS13F-HBH construct is under expressed compared to endogenously expressed KHDRBS1 at all time points. **(b)** Comparison of total number of crosslinking events as in Fig. 2b, combined into single plot (Pearson correlation 0.997, n=30, p-value $< 2.2e\text{-}16$). **(c)** Number of crosslinking events located on genomic target classes for three uvCLAP background controls in human. Each crosslinking event was assigned to the leftmost matching category. **(d)** Number of crosslinking events located on genomic target classes for two uvCLAP background controls in Drosophila. Each crosslinking event was assigned to the leftmost matching category. **(e)** Number of crosslinking events located on genomic target classes for two uvCLAP background controls in mouse. Each crosslinking event was assigned to the leftmost matching category.

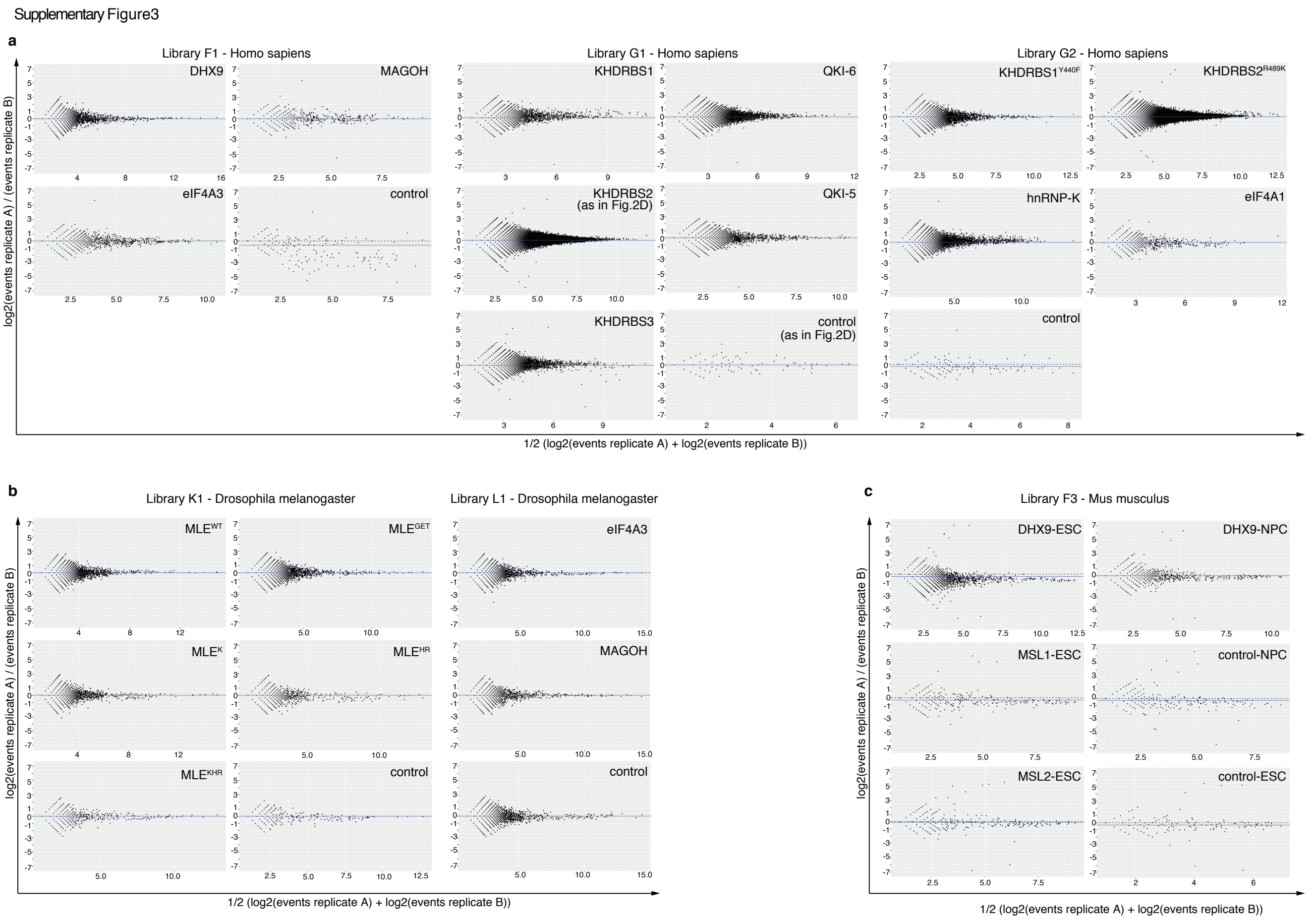

Supplementary Figure 3: MA-plots comparing crosslinking event counts for pairwise biological replicates of genomic 100 nucleotide bins covered by at least 2 crosslinking events in both replicates for **(a)** 15 uvCLAP experiments in human, **(b)** 9 uvCLAP experiments in Drosophila, and **(c)** 6 uvCLAP experiments in mouse. The median log2 fold change is indicated in blue. Related to Fig. 2c.

Supplementary Figure 4

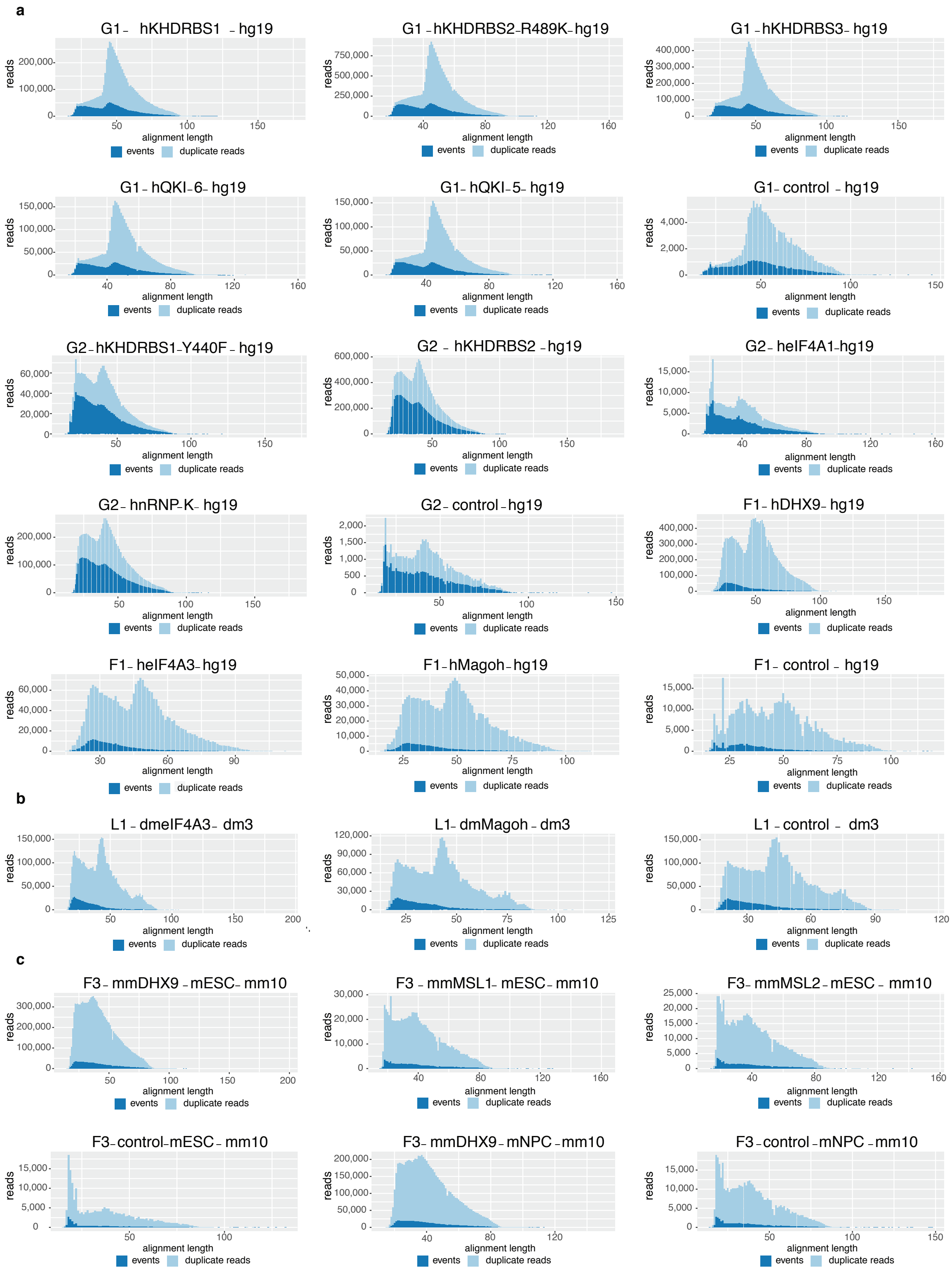

Supplementary Figure 4: Number of reads categorized as crosslinking events and PCR duplicates dependent on alignment length as proxy for cDNA insert size for **(a)** 15 uvCLAP experiments in human, **(b)** 3 uvCLAP experiments in Drosophila, and **(c)** 6 uvCLAP experiments in mouse. Related to Fig. 2e.

Supplementary Figure 5

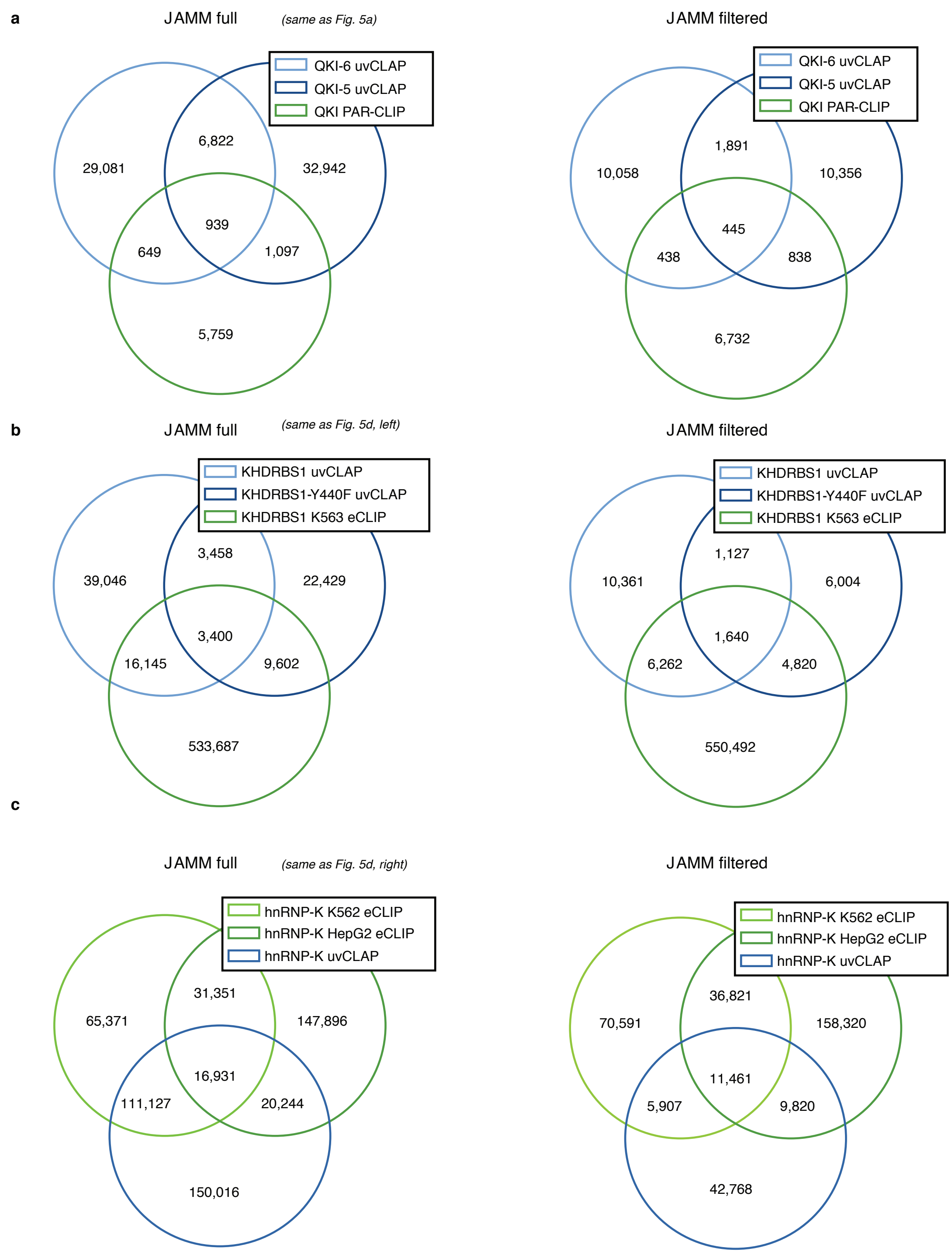

Supplementary Figure 5: Overlaps for full and filtered JAMM peaks between **(a)** QKI-5 uvCLAP, QKI-6 uvCLAP and QKI PAR-CLIP peaks; **(b)** KHDRBS1 uvCLAP, KHDRBS1Y440F uvCLAP and KHDRBS1 K564 eCLIP peaks; and **(c)** hnRNPK uvCLAP, hnRNPK K562 eCLIP and hnRNPK HepG2 eCLIP peaks. Related to Fig. 5a-d.

Supplementary Figure 6

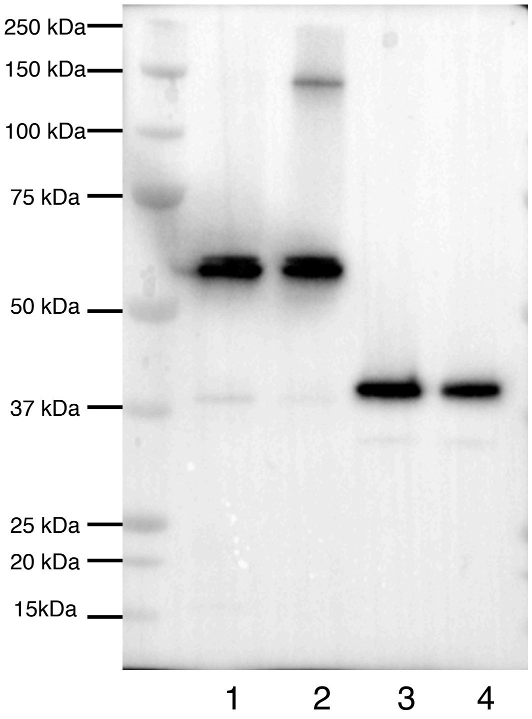

Supplementary Figure 6: Uncropped image of the blot presented in Fig. 1c.

experiment	read pairs	mapped read pairs	percent mapped	uniquely mapped read pairs	fraction uniquely mapped	crosslinking events	avg reads per event
F1 control repA	1,307,646	477,654	36.53%	172,534	36.12%	14,083	12.25
F1 control repB	4,568,976	1,166,107	25.52%	369,663	31.70%	32,371	11.42
F1 hDHX9 repA	41,315,813	31,567,521	76.41%	9,158,346	29.01%	692,429	13.23
F1 hDHX9 repB	41,325,930	31,428,779	76.05%	9,187,391	29.23%	686,441	13.38
F1 heIF4A3 repA	4,921,062	3,251,957	66.08%	1,242,806	38.22%	115,368	10.77
F1 heIF4A3 repB	5,034,306	3,467,946	68.89%	1,318,211	38.01%	120,461	10.94
F1 hMagoh repA	3,673,447	2,193,846	59.72%	908,694	41.42%	64,697	14.05
F1 hMagoh repB	3,555,747	2,152,408	60.53%	851,919	39.58%	62,851	13.55
F3 control-mESC repA	1,341,909	290,047	21.61%	112,698	38.86%	11,239	10.03
F3 control-mESC repB	1,505,169	342,932	22.78%	120,261	35.07%	12,284	9.79
F3 control-mNPC repA	2,036,638	590,303	28.98%	213,732	36.21%	20,939	10.21
F3 control-mNPC repB	2,445,617	807,502	33.02%	284,625	35.25%	27,328	10.42
F3 mmDHX9-mESC repA	13,797,220	10,731,484	77.78%	4,794,563	44.68%	438,295	10.94
F3 mmDHX9-mESC repB	22,331,283	18,091,154	81.01%	8,407,634	46.47%	765,502	10.98
F3 mmDHX9-mNPC repA	9,295,682	7,416,537	79.78%	3,762,721	50.73%	331,254	11.36
F3 mmDHX9-mNPC repB	10,043,288	7,941,592	79.07%	4,090,946	51.51%	360,292	11.35
F3 mmMSL1-A1-mESc repA	2,436,943	1,012,377	41.54%	389,426	38.47%	35,508	10.97
F3 mmMSL1-A1-mESc repB	3,527,053	1,409,099	39.95%	541,164	38.40%	49,584	10.91
F3 mmMSL2-G5-mESc repA	2,763,342	946,524	34.25%	345,839	36.54%	33,214	10.41
F3 mmMSL2-G5-mESc repB	2,920,665	1,114,303	38.15%	408,648	36.67%	38,547	10.60
G1 control repA	264,679	150,356	56.81%	87,835	58.42%	23,320	3.77
G1 control repB	261,108	135,030	51.71%	79,590	58.94%	21,155	3.76
G1 hKHDRBS1 repA	8,421,328	5,861,206	69.60%	3,602,087	61.46%	942,495	3.82
G1 hKHDRBS1 repB	6,224,221	4,986,412	80.11%	3,129,895	62.77%	827,381	3.78
G1 hKHDRBS2-R489K repA	19,941,764	16,372,513	82.10%	10,938,476	66.81%	2,903,269	3.77
G1 hKHDRBS2-R489K repB	23,049,088	15,059,914	65.34%	10,108,441	67.12%	2,690,138	3.76
G1 hKHDRBS3 repA	9,742,233	7,942,689	81.53%	5,505,964	69.32%	1,408,224	3.91
G1 hKHDRBS3 repB	8,912,187	7,083,910	79.49%	4,872,189	68.78%	1,251,194	3.89
G2 control repA	171,772	54,353	31.64%	26,147	48.11%	12,630	2.07
G2 control repB	200,107	74,607	37.28%	35,349	47.38%	16,263	2.17
G2 hKHDRBS2 repA	20,259,011	16,519,787	81.54%	9,371,716	56.73%	4,808,843	1.95
G2 hKHDRBS2 repB	18,766,041	15,475,107	82.46%	8,772,819	56.69%	4,490,947	1.95
G2 heIF4A1 repA	922,454	589,489	63.90%	162,724	27.60%	79,913	2.04
G2 heIF4A1 repB	1,025,765	643,082	62.69%	174,682	27.16%	84,031	2.08
G2 hnRNP-K repA	10,530,351	8,943,992	84.94%	4,778,058	53.42%	2,256,679	2.12
G2 hnRNP-K repB	9,360,508	7,994,570	85.41%	4,153,006	51.95%	1,972,476	2.11
K1 control repA	201,994	105,162	52.06%	71,522	68.01%	42,975	1.66
K1 control repB	248,710	115,501	46.44%	78,032	67.56%	46,905	1.66
K1 dmMLE-wt repA	1,069,726	822,726	76.91%	527,632	64.13%	332,817	1.59
K1 dmMLE-wt repB	1,042,834	799,808	76.70%	515,526	64.46%	325,564	1.58
L1 control repA	5,352,474	3,652,042	68.23%	2,376,439	65.07%	251,258	9.46
L1 control repB	6,376,992	3,967,611	62.22%	2,605,568	65.67%	256,890	10.14
L1 dmeIF4A3 repA	4,996,191	3,381,858	67.69%	2,318,915	68.57%	249,923	9.28
L1 dmeIF4A3 repB	5,544,132	3,699,513	66.73%	2,539,095	68.63%	267,781	9.48
L1 dmMagoh repA	4,082,965	2,690,605	65.90%	1,724,312	64.09%	194,246	8.88
L1 dmMagoh repB	4,056,552	2,715,860	66.95%	1,770,807	65.20%	197,389	8.97

Supplementary Table 1: uvCLAP mapping statistics.

library	pulldown	genome	median foldchange
G1	hKHDRBS1	hg19	1.00
G1	hKHDRBS2-R489K	hg19	1.00
G1	hKHDRBS3	hg19	1.00
G1	hQKI-A	hg19	1.00
G1	hQKI-B	hg19	1.00
G1	control	hg19	1.00
K1	dmMLE-GET	dm3	1.00
K1	dmMLE-HR	dm3	1.00
K1	dmMLE-K	dm3	1.00
K1	dmMLE-KHR	dm3	1.00
K1	dmMLE-wt	dm3	1.00
K1	control	dm3	1.00
G2	heIF4A1	hg19	1.00
G2	hKHDRBS1-K440F	hg19	1.00
G2	hKHDRBS2	hg19	1.00
G2	hnRNP-K	hg19	1.00
G2	control	hg19	0.80
L1	dmeIF4A3	dm3	1.00
L1	dmMagoh	dm3	1.00
L1	control	dm3	1.00
F3	mmDHX9-mNPC	mm10	1.00
F3	control-mNPC	mm10	0.83
F3	mmDHX9-mESC	mm10	0.80
F3	mmMSL1-mESC	mm10	0.80
F3	mmMSL2-mESC	mm10	0.95
F3	control-mESC	mm10	0.80
F1	hDHX9	hg19	1.00
F1	heIF4A3	hg19	1.00
F1	hMagoh	hg19	1.00
F1	control	hg19	0.67
		median	1.00
		average	0.96

Supplementary Table 2: Median between-replicate fold changes of crosslinking events for genomic bins of 100 nucleotides.

library	pulldown	organism	fg events	bg events	fg/bg	log2(fg/bg)
F1	hDHX9	hg19	1,378,869	46,454	29.68	4.89
F1	heIF4A3	hg19	235,829	46,454	5.08	2.34
F1	hMagoh	hg19	127,548	46,454	2.75	1.46
F3	mmDHX9-mESC	mm10	1,203,797	23,523	51.18	5.68
F3	mmDHX9-mNPC	mm10	691,546	48,267	14.33	3.84
F3	mmMSL1-mESC	mm10	85,092	23,523	3.62	1.85
F3	mmMSL2-mESC	mm10	71,761	23,523	3.05	1.61
G1	hKHDRBS1	hg19	1,769,876	44,475	39.79	5.31
G1	hKHDRBS2-R489K	hg19	5,593,407	44,475	125.77	6.97
G1	hKHDRBS3	hg19	2,659,418	44,475	59.80	5.90
G1	hQKI-A	hg19	1,012,625	44,475	22.77	4.51
G1	hQKI-B	hg19	960,226	44,475	21.59	4.43
G2	heIF4A1	hg19	163,944	28,893	5.67	2.50
G2	hKHDRBS1-K440F	hg19	1,154,625	28,893	39.96	5.32
G2	hKHDRBS2	hg19	9,299,790	28,893	321.87	8.33
G2	hnRNP-K	hg19	4,229,155	28,893	146.37	7.19
K1	dmMLE-GET	dm3	465,362	89,880	5.18	2.37
K1	dmMLE-HR	dm3	244,845	89,880	2.72	1.45
K1	dmMLE-K	dm3	548,249	89,880	6.10	2.61
K1	dmMLE-KHR	dm3	161,889	89,880	1.80	0.85
K1	dmMLE-wt	dm3	658,381	89,880	7.33	2.87
L1	dmeIF4A3	dm3	517,704	508,148	1.02	0.03
L1	dmMagoh	dm3	391,635	508,148	0.77	-0.38

Supplementary Table 3: Foreground to background ratios (replicates combined) for 23 uvCLAP experiments. Related to Fig. 2d.

library	pulldown	genome	JAMM peaks	no background within 50 nt	percent no background within 50 nt
F1	hDHX9	hg19	41,756	40,122	96.09%
F1	heIF4A3	hg19	5,004	4,369	87.31%
F1	hMagoh	hg19	2,299	1,827	79.47%
F3	mmDHX9-mESC	mm10	40,695	40,230	98.86%
F3	mmDHX9-mNPC	mm10	14,905	14,428	96.80%
F3	mmMSL1-mESC	mm10	479	274	57.20%
F3	mmMSL2-mESC	mm10	510	305	59.80%
G1	hKHDRBS1	hg19	67,139	62,898	93.68%
G1	hKHDRBS2-R489K	hg19	140,986	129,330	91.73%
G1	hKHDRBS3	hg19	134,764	127,265	94.44%
G1	hQKI-A	hg19	39,917	34,579	86.63%
G1	hQKI-B	hg19	44,500	41,977	94.33%
G2	heIF4A1	hg19	3,555	3,057	85.99%
G2	hKHDRBS1-Y440F	hg19	41,309	39,801	96.35%
G2	hKHDRBS2	hg19	298,135	294,198	98.68%
G2	hnRNP-K	hg19	220,004	216,607	98.46%
K1	dmMLE-GET	dm3	14,989	12,448	83.05%
K1	dmMLE-HR	dm3	5,373	4,229	78.71%
K1	dmMLE-K	dm3	11,150	9,397	84.28%
K1	dmMLE-KHR	dm3	2,454	1,634	66.59%
K1	dmMLE-wt	dm3	19,263	16,744	86.92%
L1	dmeIF4A3	dm3	12,501	3,326	26.61%
L1	dmMagoh	dm3	6,723	1,102	16.39%

Supplementary Table 4: Amount of background co-located with JAMM peaks from 23 uvCLAP experiments.

library	protein	Spearman's rank correlation rho	number of peaks
G1	hKHDRBS1	0.95140	1,109
G1	hQKI	0.95037	2,111
G1	hQKI	0.95037	785
F1	heIF4A3	0.94611	485
F1	hDHX9	0.93792	991
G2	hnRNP	0.93740	1,983
G1	hKHDRBS2	0.93190	30,304
G2	hKHDRBS2	0.93057	43,563
F1	hMagoh	0.92428	198
G1	hQKI	0.89798	2,111
G1	hQKI	0.89798	785
G1	hKHDRBS3	0.88282	1,519
G2	hKHDRBS1	0.87307	1,678
G2	heIF4A1	0.85165	300
	average:	0.91885	

Supplementary Table 5: Peak counts and pairwise Spearman correlations for PEAKachu peaks.

library	protein	rRNA	tRNA	snoRNA	snRNA	lincRNA	misc_ncRNA	pseudogene	protcod_as	3UTR	5UTR	exon	intron	antisense	intergenic
F1	hDHX9	19	205	162	27	970	671	350	542	2,443	923	1,455	29,811	992	3,186
F1	heIF4A3	7	303	173	39	94	106	45	24	854	630	2,087	295	87	260
F1	hMagoh	8	215	109	30	58	66	13	15	312	218	954	131	47	123
G1	hKHDRBS1	15	212	102	33	2,035	654	342	953	10,968	890	1,737	43,511	1,200	4,487
G1	hKHDRBS2-R489K	9	117	74	19	3,398	1,260	976	1,622	46,374	2,835	9,256	60,349	3,601	11,096
G1	hKHDRBS3	12	160	102	45	3,183	1,154	509	1,349	14,883	871	1,615	101,307	1,826	7,748
G1	hQKI-A	12	136	50	39	807	455	194	378	20,471	903	2,596	10,525	754	2,597
G1	hQKI-B	12	162	55	65	1,142	479	176	580	3,425	345	526	33,635	716	3,182
G2	heIF4A1	6	154	86	15	97	66	26	24	347	1,585	574	147	133	295
G2	hKHDRBS1-K440F	11	195	107	14	893	378	265	363	25,339	1,513	4,417	4,630	785	2,399
G2	hKHDRBS2	16	266	180	57	6,760	2,771	1,946	3,417	52,996	5,185	17,865	177,385	6,761	22,530
G2	hnRNP-K	26	304	212	71	4,931	2,884	1,097	3,269	19,086	5,948	9,058	148,914	5,504	18,700

library	protein	rRNA	snoRNA	snRNA	ncRNA	tRNA	3UTR	5UTR	exon	intron	antisense	intergenic
K1	dmMLE-GET	4	45	6	190	18	2,323	885	1,969	5,879	1,067	2,603
K1	dmMLE-HR	1	51	9	111	36	526	378	543	2,514	366	838
K1	dmMLE-K	4	66	7	173	36	927	710	1,084	5,511	898	1,734
K1	dmMLE-KHR	2	96	11	87	17	372	355	523	572	109	310
K1	dmMLE-wt	3	63	6	206	34	1,353	1,045	1,478	10,689	1,455	2,931
L1	dmeIF4A3	22	141	13	106	33	1,336	1,960	7,184	914	190	602
L1	dmMagoh	24	163	15	70	92	1,157	1,190	2,561	704	176	571

library	protein	rRNA	tRNA	snoRNA	snRNA	lincRNA	misc_ncRNA	pseudogene	3UTR	5UTR	exon	intron	antisense	intergenic
F3	mmDHX9-mESC	2	173	127	22	458	5	48	2,053	1,113	642	32,861	693	2,498
F3	mmDHX9-mNPC	2	72	85	11	210	4	14	1,373	585	508	11,165	159	717
F3	mmMSL1-mESC	1	30	64	10	18	6	1	34	57	36	69	45	108
F3	mmMSL2-mESC	2	29	51	8	21	5	1	38	65	45	68	39	138

Supplementary Table 6: Number of crosslinking events located on target classes. Related to Fig.s 3 and 4a,b and Supplementary Fig. 2c-e.

id	tag
5’-tag 1	AGTCA
5’-tag 2	GAGGT
5’-tag 3	ATCGT
5’-tag 4	GTTCT
5’-tag 5	TACCT
5’-tag 6	AGAAT
5’-tag 7	ACTTG
5’-tag 8	GTATG
5’-tag 9	CGGCG
5’-tag 10	TCACG
5’-tag 11	GCCAG
5’-tag 12	CTCTC
5’-tag 13	TGATA
5’-tag 14	GCTGA
5’-tag 15	CTACA

Supplementary Table 7: Selected uvCLAP 5’-tags.
